# Impact of CD 28, CD86, CTLA-4 and PD-1 genes polymorphisms on acute renal allograft rejection and graft survival among Egyptian recipients

**DOI:** 10.1038/s41598-024-52195-0

**Published:** 2024-01-24

**Authors:** Moatasem Elsayed Ghoneim, Hussein Sheashaa, Ehab Wafa, Amira Awadalla, Asmaa E. Ahmed, Mohamed Sobh, Ahmed Abdulrahman Shokeir

**Affiliations:** 1 Nephrology and Transplant Unit, Urology and Nephrology Center, Mansoura University, Mansoura, Egypt; 2 Department of Urology, Urology and Nephrology Center, Mansoura University, Mansoura, Egypt; 3 Center of Excellence for Genome and Cancer Research, Urology and Nephrology Center, Mansoura University, Mansoura, Egypt; 4 Genetics Research Unit, Faculty of Medicine, Delta University for Science and Technology, Gamasa, Egypt

**Keywords:** Biological techniques, Genetics, Immunology

## Abstract

To study the impact of four gene polymorphisms on acute renal allograft rejection (AR) and graft survival among Egyptian population. These 4 gene polymorphisms include: (1) CD 28 (rs3116496), (2) CD86 (rs1129055), (3) CTLA-4 (rs3087243), (4) PD-1 (rs2227982). This is a non-concurrent cohort study including 50 kidney transplant recipients diagnosed histopathologically as (AR) [study group] and another 50 matched allograft recipients without AR [control group]. Blood samples were taken from both groups and subjected to genotyping for the selected four genetic polymorphisms by TaqMan genotyping assay. The difference in genotypic distribution of CD 28: rs3116496 and CD86: rs1129055 wasn't statistically significant between the study and control groups (P = 0.22 and 0.33 respectively) and also both polymorphisms had no effect on graft survival (P = 0.36 and 0.74 respectively) while the addition of C allele to IVS3 +17T/C polymorphism in CD28 gene showed a protective effect against AR (P = 0.03). CTLA-4: rs3087243 AG genotype showed a protective effect against AR as it was more frequent in no rejection group compared to those with AR (P = 0.001) with a statistically significant impact on graft survival (P < 0.001), while PD-1: rs2227982 AG genotype was equally distributed between both groups (variant of unknown significance). There was no detected association between CD86 polymorphism: rs1129055 and CD 28 polymorphism: rs3116496 with the development of AR. However, C allele of CD 28 IVS3 +17T/C polymorphism and CTLA-4 polymorphism: rs3087243AG genotype both demonstrated a protective effect against AR.

## Introduction

Inspite of advancement in immunosuppressive regimens, acute rejection (AR) is still the biggest challenge after Kidney transplantation (KT)^1^. T-cell mediated immune response is crucial in KT and plays an important role in allograft rejection. Proper action of T cells requires two opposing signals, the first is for T cell activation that starts with the co-stimulatory Cluster of Differentiation 28 (CD28) on naïve T cell surface binding with its ligand CD86 on antigen presenting cell (APC). This co-stimulatory signal competes with the second inhibitory signal of cytotoxic T lymphocyte-associated protein 4 (CTLA-4) on activated T cell binding with its ligand CD86 on APC (CTLA-4-CD86) to inhibit T-cell activation and proliferation^2^. Single nucleotide polymorphisms (SNPs) located in these genes have been extensively studied with contradictory results.

The rs3116496 polymorphism of the CD28 gene located on chromosomal region 2q33.2 results in a T/C substitution at position 17 in the third intron^3^. Previous researchers have reported the association between this SNP and susceptibility to AR^4,5^ while others showed lack of this association^6^.

The rs1129055 polymorphism of the CD86 gene located on chromosomal region 3q21.33^7^, introduces a possible phosphorylation site in the cytoplasmic tail of CD 86^8^ so, affecting APC signal transduction. Previous researchers has linked this CD86 +1057G/A polymorphism to a decreased incidence of AR^9,10^, while others showed lack of this association^11^.

CTLA-4, also known as CD152, is a trans-membrane glycoprotein receptor located on chromosomal region 2q33.2 and is composed of four exons^12^. It is expressed on surface of activated T cells and acts as a regulator of the immune response^13^ to inhibit cytokine release and down-regulate T cell proliferation^14^. Some studies showed association between CTLA-4 polymorphisms and AR and graft survival^15,16^ while other didn᾽t^17,18^.

Programmed cell death 1 (PD-1), is an inhibitory receptor for the suppression of T-cell activation is encoded by PDCD1 gene located on chromosome 2q37.3 and thus abnormal expressions of PD-1 protein might cause disturbance in self-tolerance^19^.

The disparities in outcomes between studies could be attributed to their small sample size, with just a small number of patients participating^4^. Other considerations include the prior research subjects' heterogeneity in terms of race, which may alter genetic expression^20^, the type of donors whether living or deceased^21^ and the type of induction therapy^22^.

In this study, we tried to overcome the limitations of previous studies and examined the impact of selected four gene polymorphisms on AR and graft survival among a cohort of Egyptian recipients. These 4 gene polymorphisms include: (1) CD 28 (rs3116496), (2) CD86 (rs1129055), (3) CTLA-4 (rs3087243), (4) PD-1 (rs2227982). The knowledge about these genetic polymorphisms and their association with AR may add a benefit in categorizing renal transplant candidates according to their risk of rejection and thus choosing the best human leucocyte antigen (HLA) matching, providing them more meticulous follow up, avoiding steroid free regimen or offering stronger induction therapy, aiming at better and longer preservation of the graft.

## Materials and methods

### Patients

**Figure 1:**
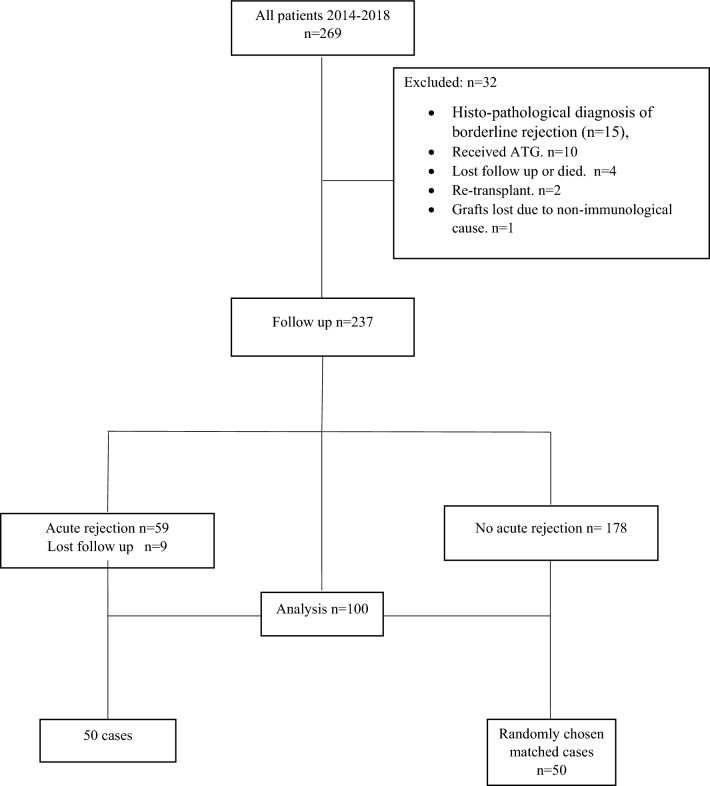
Flowchart of study design.

This a non-concurrent cohort study of 269 consecutive patients who underwent a living-donor KT at our center in a time period between January 2014 and December 2018. Of these, 32 patients were excluded from the study because of histopathological diagnosis of borderline rejection (n = 15), received thymoglobulin as an induction therapy (n = 10), lost follow up or died (n = 4), re-transplantation (n = 2) and graft loss due to non-immunological causes (n = 1). Of the remaining 237 patients, 59 consecutive patients experienced histologically proven AR, nine patients were not available for follow up and the remaining 50 patients were enrolled in the study and were considered as study group. Another 50 renal allograft recipients who didn`t experience AR during the same period were chosen using propensity matching score and included in our study as a control group. The study and control groups were matched regarding the timing of transplantation, demographics and all variables except experiencing AR. A flowchart of study design is shown in Fig. 1.

A complete pre-transplant clinical history including age, sex, primary kidney disease, duration of dialysis, previous blood transfusion or pregnancies together with full clinical, laboratory and ultrasound assessment were obtained from all kidney transplant candidates^23^.

Sample size: Sample size was calculated using G-power program with effect size = 0.5; α. Error = 0.05 and power 80% and it was equal to 100 according to population size in a previous study by Guo et al.^24^.

We included patients who have at least one HLA class II DR matching with the donor, received monoclonal anti-C25 antibody (Basiliximab) as an induction therapy, maintained either on dual [tacrolimus (Tac) and mycophenolate mofetil (MMF)] or triple immunosuppressive regimens [steroid, Tac, MMF] according to the absence (dual) or presence (triple) of panel reactive antibody (PRA) and had a proven histopathological diagnosis of AR that was graded according to BANFF classification. We included only those with acute cellular rejection grade ≥ 1 while we excluded those with high immunological risks defined as zero DR mismatch, positive crossmatch, second kidney transplantation, allograft biopsy proven chronic rejection or any pathology other than AR and non-adherent patients to immunosuppressive drugs.

AR group (study group) was defined by clinical diagnosis (elevated serum creatinine > 30% of basal line in the absence of other pathology including infection, urinary tract obstruction, allograft artery stenosis or calcineurin toxicity and responded to treatment by immunosuppression) and was confirmed by a graft biopsy showing the morphological pattern of AR while no rejection group (control group) was defined by absence of past history of biopsy proven acute allograft rejection, normal baseline serum creatinine and bland urine analysis. Blood samples were collected from the study and control groups for genotyping assay.

Our primary outcome was defining the association between gene polymorphisms of CD 28 rs3116496 SNP, CD 86 rs1129055 SNP, CTLA-4 rs3087243 SNP and PD-1 rs2227982 SNP with the development of AR. The secondary outcome was studying the impact of these genetic polymorphisms on graft survival. Follow up was carried out every one to two months in the first year post-transplantation and then every 3 months in the following 4 years in the outpatient clinic by testing serum creatinine, tacrolimus trough level and graft ultrasound if needed. The patients were admitted to our center in case of unexplained rising of serum creatinine with normal sonography. The duration of follow up ranged between 12 and 101 months median peroid of 60.39 months.

### Genotyping assay

Three millimeters of EDTA-anticoagulated peripheral whole blood were collected from AR and no rejection groups. DNA was extracted from all blood samples using GeneJET Whole Blood Genomic DNA Purification Mini Kit (cat no. K0781). The purity and quantity of extracted DNA was evaluated by Nanodrop 2000c. The predesigned TaqMan^®^ SNP Genotyping assays were used for the analysis of CD86 (rs1129055, C_7504226_10), CD28 (rs3116496, C_25922478_10), PD-1(rs2227982, C_57931287_10) and CTLA4 (rs3087243, C_3296043_10) according to the manufacture. Ten ng of DNA was mixed with TaqMan Genotyping Master Mix (Applied Biosystems) and was analyzed using StepOne plus Real Time PCR (Applied Biosystems, Foster City, CA, USA). The PCR conditions were an initial cycle at 60 °C for 30 s followed by one cycle at 95 °C for 10 min and finally 40 cycles at 95 °C for 15 s and at 60 °C for 1 min.

### Statistical analyses

Qualitative variables were expressed as frequency and percentage and compared by using the chi-square test or Fisher exact test as appropriate. Quantitative variables were expressed as mean and standard deviation (SD) for normally distributed variables and as median and range for non-normally distributed variables. Student`s t-Test and Mann–Whitney test were used for comparison between the 2 groups as appropriate. A probability of P < 0.05 was considered statistically significant, Survival was estimated by Kaplan–Meier method, and survival curves were compared with log-rank test. The strength of the association between genotypes or alleles in each group was estimated by the calculation of the odds ratios (OR) and 95% confidence intervals (CI). The chi-square test was used for the comparison of the distribution of genotype frequencies among the groups tested with Hardy–Weinberg equilibrium.

### Ethical approval

Ethical approval was given by our local Institutional Research Board of Mansoura University (code: MD.20.04.321.R1). Authors ensure that the work described has been carried out in accordance with The code of Ethics of the World Medical Association (Declaration of Helsinki) for experiments involving humans. Fully informed consent to participate in the study was taken.

## Results

### Patients

Both groups were comparable regarding recipient age and sex, recipient blood group, consanguinity, donor age and sex, original kidney disease and pre-transplant dialysis duration and medical disorders. Also there was no-statistically significant difference regarding the degree of HLA mismatching, and pre-transplant anti- HLA antibodies. The demographic and clinical characteristics of the 100 kidney transplant recipients are shown in Table 1.

**Table 1:** Demographics and clinical characteristics of the kidney transplant cohort.

	Acute rejection (n = 50)	No rejection (n = 50)	P value
Recipient sex, no. (%)			
Male	38 (76)	35 (70)	0.49*
Female	12 (24)	15 (30)	
Recipient age, (years), mean ± SD	26.04 ± 10.4	23.7 ± 7.54	0.42***
Recipient blood groups, no. (%)			
A	24 (48)	19 (38)	0.74*
B	10 (20)	11 (22)	
AB	11 (22)	15 (30)	
O	5 (10)	5 (10)	
Donor sex, no. (%)			
Male	22 (44)	19 (38)	0.54*
Female	28 (56)	31 (62)	
Donor age (years), mean ± SD	42.70 ± 9.15	40.02 ± 9.91	0.29***
Consanguinity, no. (%)			
Related	42 (84)	46 (92)	0.22*
Unrelated	8 (16)	4 (8.0)	
Original kidney disease, no. (%)			
Unknown	36 (72)	27 (54)	0.089**
Glomerulonephritis	8 (16)	8 (16)	
Hereditary	2 (4.0)	2 (4.0)	
Urological	2 (4.0)	10 (20)	
Nephrosclerosis	1 (2.0)	0 (0)	
Interstitial nephritis	1 (2.0)	3 (6.0)	
Pretransplant medical disorders, no. (%)			
Hypertension	29 (58)	23 (48)	0.32*
HCV infection	8 (16)	7 (14)	0.87*
HBV infection	1 (2.0)	1 (2.0)	0.99**
HIV infection	0 (0)	0 (0)	
Blood group compatibility, no. (%)			
Identical	36 (72)	38 (76)	0.65*
Different	14 (28)	12 (24)	
Number of pre-transplant blood transfusions (unit), mean ± SD	1.8 ± 1.25	1.55 ± 0.71	0.25***
Dialysis type, no. (%)			
No	0 (0)	1 (2)	0.99**
Hemodialysis	49 (98)	49 (98)	
Peritoneal dialysis	1 (2)	0 (0)	
Dialysis duration (months), mean ± SD	27.85 ± 5.73	29.25 ± 4.57	0.198***
HLA class I mismatch, no. (%)			
0 MM	4 (8.0)	7 (12)	0.82**
1 MM	3 (6.0)	4 (8)	
2 MM	31 (62)	29 (58)	
3 MM	7 (12)	3 (8.0)	
4 MM	6 (12)	7 (14.0)	
HLA class II mismatch, no. (%)			
0 MM	5 (10)	2 (16)	0.37**
1 MM	45 (90)	2 (84)	
Positive anti-HLA antibodies, no. (%)	3 (6)	0 (0)	0.27**
Ischemia time (minutes), mean ± SD	48.7 ± 15.4	46.2 ± 9.9	0.34***
Time to diuresis, no. (%)			
Immediate	46 (92)	49 (98)	0.36**
Delayed	4 (8.0)	1 (2.0)	
Maintenance therapy, no. (%)			
Triple	31 (62)	30 (60)	0.84*
Dual	19 (38)	20 (40)	
Post-transplant complications, no (%)			
Living on dialysis	13 (26)	0 (0)	0.001**
Acute tubular injury	9 (18)	1 (2.0)	0.008**
Diabetes	4 (8.0)	4 (8.0)	0.99**
Hypertension	45 (90)	23 (46)	0.001*
Hepatic impairment	2 (4.0)	1 (2.0)	0.99**
Bacterial infection	9 (38)	1 (2)	0.001**
CMV infection	5 (10)	1 (2)	0.20**

*Chi-square test.

**Fisher exact test.

***Student t-Test.

### Gene polymorphism and acute allograft rejection

#### CD 28 gene rs3116496 polymorphism and acute allograft rejection

Rejection patients were at H-W equilibrium as regard *rs3116496 polymorphism (p* = *0.2).* The difference in distribution of (CD28 IVS3 +17T/C) genotypes (TT, CT and CC) was not statistically significant between both AR and no rejection group (P = 0.224). The frequency of TT genotype was higher in the AR group (70%) than in those with no rejection (54%), but the difference wasn`t statistically significant in the dominant model (TT Vs CT + CC) (P = 0.149). CC genotype was more frequent in the no rejection group (38%) than in AR group (26%), but also the difference wasn`t statistically significant in the recessive model (TT + CT Vs CC) (P = 0.647). There was an increase of the C allele frequency among the patients who did not developed an AR (42%), compared to the kidney recipients with AR (28%) with a difference of statistical significance (P = 0.038) (Table 2).

**Table 2:** Distribution of genotypes and allele frequencies among AR and no rejection group.

Gene polymorphism No. (%)	Genotypes and alleles	Acute rejection (n = 50)	No rejection (n = 50)	OR (95% CI)	P value*
CD 28 IVS3 + 17T/C rs3116496					
Genotypes	TT	35 (70)	27 (54)		0.244
	CT	2 (4.0)	4 (8.0)	0.53 (0.22–2.25)	
	CC	13 (26)	19 (38)	0.39 (0.05- 2.13)	
		HWEp = 0.2	HWEp = 0.2		
Dominant model					
TT Vs CT + CC	CT and CC	15 (30)	23 (46)	1.9 (0.8–4.5)	0.149
	TT	35 (70)	27 (54)		
Recessive model					
CT + TT Vs CC	CT and TT	48 (96.0)	46 (92.0)	2.08 (0.36–11.9)	0.674
	CC	2 (4.0)	4 (8.0)		
Alleles					
C Vs T	T allele	72 (72)	58 (58)	0.53 (0.29–0.9)	0.038
	C allele	28 (28)	42 (42)		
CD86 +1057G>A rs1129055					
Genotypes	G/G	36 (72)	33 (66)		0.339
	A/G	14 (28)	15 (30)	0.86 (0.36–2.04)	
	A/A	0 (0)	2 (4.0)	0.3 (0.2–1.4)	
		HWEp = 0.24	HWEp = 0.24		
Recessive model					
AG + GG Vs AA	AG and GG	50 (100)	48 (96.0)	5.2 (0.24–111.2)	0.475
	AA	0 (0.0)	2 (4.0)		
Dominant model					
GG Vs AG + AA	GG	36 (72.0)	33 (66.0)	1.3 (0.56–3.1)	0.665
	AG and AA	14 (28.0)	17 (34.0)		
Alleles					
A Vs G allele	G allele	86 (86)	81 (81)	0.69 (0.32–1.4)	0.34
	A allele	14 (14)	19 (19)		
CTLA-4 + 6230 A/G rs3087243					
Genotypes	G/G	9 (18)	0 (0.0)		0.001
	A/G	31 (62)	46 (92)	0.9 (0.6–3.1)	
	A/A	10 (20)	4 (8)	3.71 (1.13- 14.5)	
		HWEp = 0.04	HWEp = 0.04		
Recessive model					
AA.vs.AG + GG	AG and GG	40 (80.0)	46 (92.0)	2.87 (0.8–9.8)	0.150
	AA	10 (20.0)	4 (8.0)		
Dominant model					
GG vs AG + AA	GG	9 (18.0)	0 (0.0)	23.12 (1.3–409)	0.005
	AG and AA	41 (82.0)	50 (100.0)		
Alleles					
A Vs G allele	G allele	49 (49)	46 (46)	0.88 (0.5–1.5)	0.6
	A allele	51 (51)	54 (54)		
PD-1 rs2227982 genotypes	G/G	0 (0)	0 (0)		
	A/G	50 (100)	50 (100)		
	A/A	0 (0)	0 (0)		

*Logistic regression.

#### CD86 gene rs1129055 polymorphism and acute allograft rejection

Rejection patients were at H-W equilibrium as regard *rs3116496 polymorphism (p* = *0.245).* The difference in distribution of (CD86 +1057G>A) genotypes (GG, AG and AA) and alleles (A and G alleles) frequencies was not statistically significant between both AR and no rejection groups (P = 0.339 and 0.34 respectively). GG genotype was more frequent in the AR group (72%) than in those with no rejection (66%), but the difference wasn`t statistically significant in the dominant model (P = 0.665). AA genotype was absent in the AR group. The frequency of “A” allele was higher among patients who did not developed an AR (19%), compared to those with AR (14%) (P = 0.34), but again the difference wasn`t statistically significant in the recessive model (AG + GG Vs AA) (P = 0.475) (Table 2).

#### CTLA-4 gene (rs3087243) polymorphism and acute allograft rejection

Rejection patients were not at H-W equilibrium as regard *rs3116496 polymorphism (p* = *0.04).* The difference in distribution of (CTLA-4 +6230 A/G) genotypes (GG, AG and AA) was statistically significant between both AR and no rejection groups and the frequency of AG genotype was significantly higher in the no AR group (92%) than in those with AR (62%) (P = 0.001). Also there was a statistically significant difference in the dominant model (AG + AA.vs.GG) (P = 0.005), while it was non-significant in the recessive model (AA. vs. AG + GG) (P = 0.150). The difference in distribution of (CTLA-4 +6230 A/G) alleles (A and G alleles) wasn᾽t statistically significant between both groups (P = 0.6). However, “A” allele was more frequent among no rejection group (54%), compared to the kidney recipients with AR (46%) [OR 95%CI = 0.88 (0.5–1.5)] (Table 2).

#### PD-1 gene (rs2227982) polymorphism and acute allograft rejection

The difference in distribution of PD-1 (rs2227982) genotypes showed equal distribution of AG genotype between both AR and no rejection groups (variant of unknown significance) (Table 2).

### Gene polymorphism and graft survival

#### CD 28 gene rs3116496 polymorphism and graft survival

**Figure 2:**
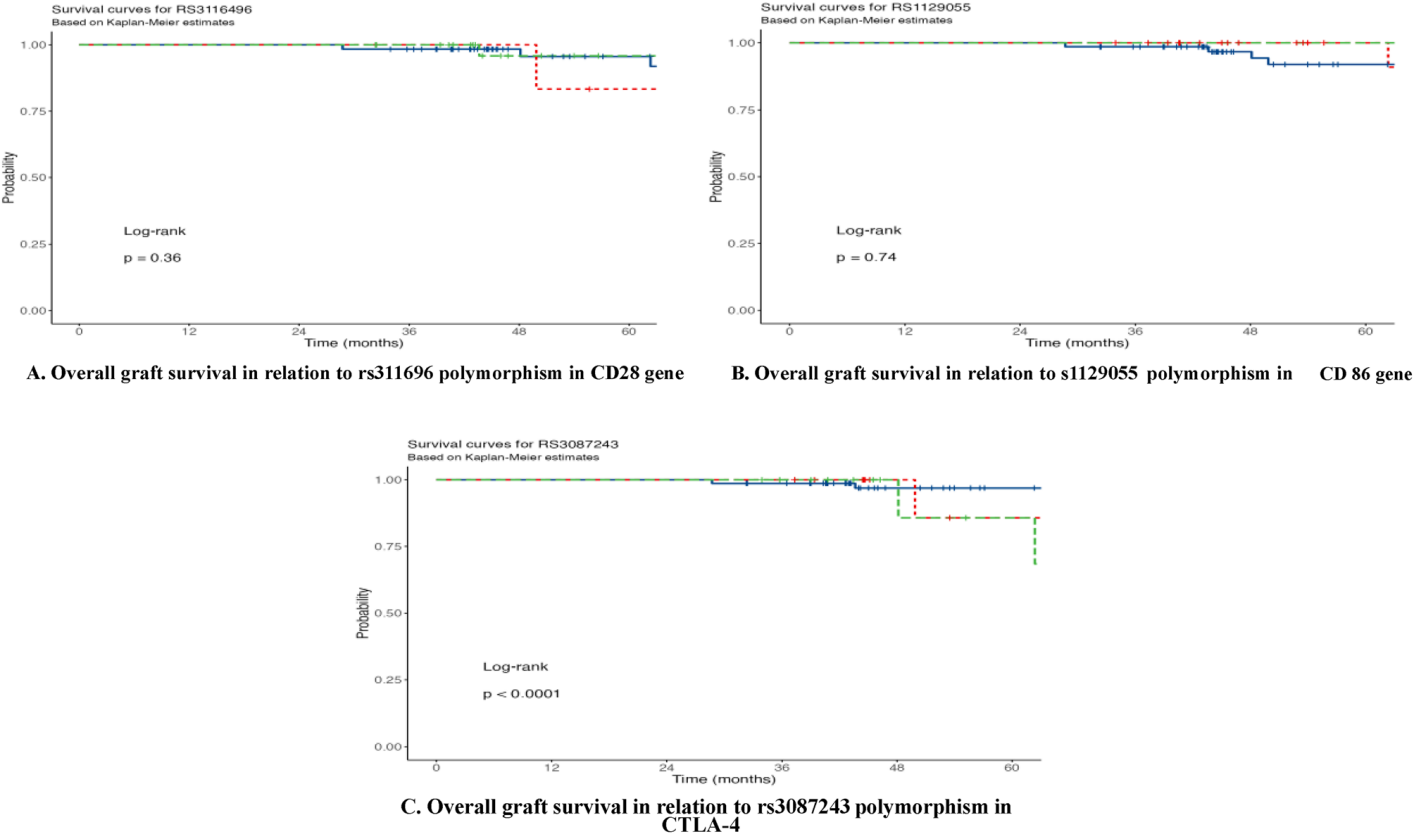
Overall graft survival.

Genotypes polymorphism (TT, CT and CC) of (CD28 IVS3 +17T/C) had no significant impact on graft survival. All our patients enjoyed a well-functioning graft at 1 year of follow up while the percentage of 3- year graft survival was 98.4%, 100% and 100% among TT, CT and CC genotypes, respectively and reached 95.6%, 95.8% and 83.3% among the same genotypes, respectively at 5 years of follow up with difference of no statistical significance (P = 0.36) (Fig. 2A).

#### CD86 gene rs1129055 polymorphism and graft survival

Genotypes polymorphism (GG, AG and AA) of (CD86 +1057G > A) showed no significant effect on the graft survival. The highest survival benefit was detected in mutant genotype (AA genotype) and heterozygous genotype (AG genotype) with a 100% graft survival for both genotypes after 3 and 5 years of follow up while graft survival decreased to 98,6% and 91.9% in GG genotype at 3 and 5 years, respectively, but with no significant difference (P = 0.74) (Fig. 2B).

#### CTLA-4 gene (rs3087243) polymorphism and graft survival

Genotypes polymorphism (GG, AG and AA) of (CTLA-4 gene +6230 A/G) showed significant impact on graft survival (P < 0.001). AG genotype had the best survival benefit in comparison to AA and GG genotypes with a 100% graft survival after 1 year of follow up for all genotypes (AG, AA and GG) while the percentage of graft survival among the same genotypes reached 98.6%, 100% and 100% at 3 years and 92.7%, 63.3% and 63.3% at 5 years respectively (Fig. 2C).

### Post transplant complications

There was a statistically significant difference between AR and no rejection group regarding post transplant graft failure, acute tubular necrosis, hypertension and bacterial infection, being more frequent in AR group (P = 0.001, 0.008, 0.001 and 0.001 respectively), while there was no statistically significant difference between both groups regarding post transplant diabetes, hepatic impairment and CMV infection (Table: 2).

## Discussion

T-cell mediated immune response plays an important role in allograft rejection. This includes either stimulatory signal for T cell activation by the interaction of CD28 expressed on resting T cell surface with its APC surface ligands, B7-1 (CD 80) or B7-2 (CD 86) or inhibitory signal by the interaction of cytotoxic T-lymphocyte-associated antigen 4 (CTLA4) on cell surface of activated T cell with their ligands on APC, B7-1 (CD 80) or B7-2 (CD 86). The polymorphisms located in these genes have been widely investigated to verify the association between these gene variants and AR with inconsistent results. We focused our attention in this study on associations of the four SNPs (CD 86: rs1129055, CD 28: rs3116496, CTLA-4: rs rs3087243 and PD-1: rs2227982) with acute allograft rejection and graft survival.

Our findings suggest that CD28 gene (IVS3 +17T > C) polymorphism has no relationship with AR and allograft survival. Taken together, our results came in agreement with a cohort of 314 Caucasian allograft recipients who were followed up for a median time of 97.5 months and showed no relationship between the same allele variants and AR or graft function either in univariate or multivariate analyses^6^. The same findings were obtained with regard to the lack of relationship between rs3116496—*CD28* and AR^22^. Moreover, with deceased donor transplants, the same outcome was obtained with no defined clear association between variations in CD 28 gene SNP and AR^18^. Contradictory to our results, other researchers reported a statistically significant association between CD28 IVS3 +17T/C variant in the dominant genetic model (C/C or C/T vs. T/T) and development of AR^4^. Another study reported a substantial link between IVS3 +17T/C (rs3116496: T/C) polymorphism in the CD28 gene and the development of AR that was statistically significantly higher in the carriers of C allele (with CT or CC genotype) compared to TT homozygotes^5^. Similar results were also reported by Liu and his colleagues who showed a significant role of CD28 IVS3 +17 T/C polymorphisms in kidney transplantation rejection and graft survival^25^.

Our findings suggest that CD 86 gene +1057G>A polymorphism didn`t show a statistically significant association with AR. In agreement with our results, CD 86 gene rs1129055 and CD 28 gene rs3116496 polymorphism were reported to have non-significant association with AR either in the dominant or in the recessive models ^22^. However, Han et al., reviewed three studies (234 cases and 411 controls) to define the relationship between the CD86 +1057G/A polymorphism and allograft rejection and reported a significant association between CD86 +1057G/A polymorphism and reduced AR in the dominant genetic model (A/A or A/G vs. G/G)^4^. Similar findings were published after observing a cohort of 168 kidney allograft Tunisian recipients who revealed a significant association between AA genotype and “A” allele of CD86 +1057G>A polymorphism and lower risk of AR and concluded that this polymorphism may be protective against AR^10^. In liver transplantation, +1057AA genotype and “A” allele of the CD86 gene were also reported to have a beneficial effect on reducing incidence of allograft rejection^26^.

Our results showed that AG genotype of CTLA-4 rs3087243 SNP was the most common genotype among no rejection group and proved a protective effect against AR and this finding came in agreement with the known inhibitory effect of CTLA-4 on T cell activation and so protection against kidney allograft rejection. Our combined results were similar to a cohort of 632 kidney transplant patients who showed a protection against kidney transplant rejection among carriers of the CTLA-4 +6230 G allele which is proved by detecting lower levels of soluble cytotoxic T lymphocyte antigen-4 micro RNA (sCTLA-4 mRNA)^22^. In deceased kidney transplantation, a retrospective study examining 72 deceased donor derived kidney transplant recipients who completed 6 months of follow up post transplantation, reported a significant higher incidence of CT60 A/A genotype in AR group compared with no rejection group (29.7% vs 8.6%; P = 0.035; OR = 4.51) and a higher incidence of CT60 A/G genotype in the no rejection group^27^. In contrast to our results, examining CTLA-4 rs3087243 SNP in 678 deceased donor derived kidney transplant recipients, showed non-significant association with AR^18^. Again, following the course of 167 kidney transplant recipients who received their kidneys from deceased donors revealed no differences between AR and non-AR patients in genotypes of rs3087243 either in the dominant or recessive model^28^. Similarly in a study of Turkish patients, including 34 kidney transplant recipients with AR versus 47 patients without AR and 50 cases of healthy volunteers, they didn`t find any influence of CTLA-4 CT60A/G (rs3087243) on AR^29^.

Our results showed that AG genotype of PD-1 rs2227982 SNP was equally distributed between both AR and no rejection group (variant of unknown significance). Up to our knowledge, Zolfaghari and his colleagues were the only researchers who examined this SNP in a cohort of 122 Iranian patients who were divided into 2 groups, either AR or stable graft function group and they reported non-significant difference in genotypes and alleles distribution between both groups and so non- significant impact on AR^30^.

Our explanation for this conflicting results in literature might be due to the fact that these studies were applied on different populations and races with different genetic variations and it is well established that the ethnic background may influence the gene expression and susceptibility to some medical disorders^20^. Also the type of kidney donor whether deceased or living together with the type of induction therapy may have an influence on gene expression, for example one study reported a statistically significant association between patients carrying CTLA-4 +49 AA genotype and lower AR episodes in transplanted recipients who received their kidneys from a living related donor while this association disappeared when compared to another group of transplanted recipients who received their kidneys from a cadaveric unrelated donors^21^. This observation was confirmed by other researchers who showed that in patients receiving thymoglobulin induction therapy, correlations with rejection were not identified for any SNP even for those showed initial significance^22^.

Our study is a controlled one with a relatively long follow up period and, up to our knowledge, this is the first research to be conducted on Egyptian kidney transplant population. Nevertheless, our study has some limitations including, the study design being a non-concurrent rather than a cohort study and the sample size is relatively small so, further prospective better controlled studies with larger sample sizes are recommended. Moreover, examining more SNPs are needed to determine the clinical relevance of this genetic variation with kidney allograft outcome. Until that time, genetic variations may have a clinical impact when added, hand in hand, beside other immunologic tests like HLA tissue matching, serum lymphocytotoxicity cross match, anti-HLA antibodies. These immunologic markers could be combined with other risk factors for prediction of AR such as age, sex, blood group compatibility, pre or post-transplant medical complications …etc. All these factors could be combined all together in a model of artificial intelligence for prediction of AR and determining the need for augmenting immunosuppressant drugs.

## Conclusion:

Our findings point to the CTLA-4 polymorphism rs3087243 (+6230 A/G) as a promising biomarker for kidney allograft rejection with significant impact on graft survival, particularly for TCMR Banff graded ≥ 1 while both CD 86 polymorphism rs1129055 (+1057G>A) and CD 28 polymorphism rs3116496 (IVS3 +17T/C) showed non-significant association with AR and graft survival. However, there was a statistical significant increase of the C allele frequency of CD 28 (IVS3 +17T/C) among the no rejection group (P = 0.038). PD-1 rs2227982 showed variation of unknown significance.

## Data Availability

All data generated or analyzed during this study are included in this article. Further enquires can be directed to the corresponding author.
